# Trabecular microbypass as replacement therapy in pharmacologically controlled open-angle glaucoma patients

**DOI:** 10.5935/0004-2749.20230034

**Published:** 2023

**Authors:** Ricardo Augusto Paletta Guedes, Daniela Marcelo Gravina, Vanessa Maria Paletta Guedes, Alfredo Chaoubah

**Affiliations:** 1 Universidade Federal de Juiz de Fora, Juiz de Fora, MG, Brazil; 2 Instituto de Olhos Paletta Guedes, Juiz de Fora, MG, Brazil

**Keywords:** Ophthalmologic surgical procedures, Cataract extraction, Glaucoma, open-angle, Glaucoma/therapy, Glaucoma/surgery, Procedimentos cirúrgicos oftalmológicos, Extração de catarata, Glaucoma, ângulo aberto, Glaucoma/terapia, Glaucoma/cirurgia

## Abstract

**Purpose:**

To assess the outcomes of the trabecular bypass as replacement therapy for medications in pharmacologically controlled vs. pharmacologically uncontrolled open-angle glaucoma patients.

**Methods:**

This was a retrospective study of eyes treated with first- (iStent) or second-generation (iStent *inject*) trabecular bypass. Group 1 consisted of eyes with pharmacologically controlled intraocular pressure <18 mmHg and Group 2 consisted of eyes with pharmacologically controlled intraocular pressure ≥18 mmHg. The main outcomes measured were qualified (with or without medications) and unqualified or complete (without medications) success rates at different target intraocular pressures, mean reduction (%) in medication use, and proportion of medication-free eyes.

**Results:**

The mean age was 70.4 years in Group 1 (n=105) and 68.1 years in Group 2 (n=65). Qualified success rates for intraocular pressure <18 mmHg, intraocular pressure <15 mmHg, and intraocular pressure <12 mmHg were similar between the groups (Group 1: 96.2%, 88.6%, and 32.4%, respectively; Group 2: 93.8%, 78.5%, and 21.5%, respectively; all p>0.05). Complete success rates were significantly higher in Group 1 than in Group 2: for intraocular pressure <18 mmHg (76.2% vs. 47.7%), intraocular pressure <15 mmHg (73.3% vs. 40.0%), and intraocular pressure <12 mmHg (14.3% vs. 4.6%). The mean reduction in medication use was higher in Group 1 than in Group 2. At the end of follow-up, 79.0% of eyes in Group 1 and 47.7% of eyes in Group 2 became medication-free.

**Conclusions:**

Both groups showed high qualified success rates, but eyes with baseline pharmacologically controlled intraocular pressure <18 mmHg showed higher complete success rates and greater chances of achieving no need for medications.

## Abstract (pt)

**Objetivo:**

Avaliar os resultados dos implantes de by-pass trabecular como terapia de substituição aos colírios em pacientes com glaucoma de ângulo aberto controlados com medicação vs. não controlados com medicação.

**Métodos:**

Este foi um estudo retrospectivo de olhos submetidos a cirurgia de implante de by-pass trabecular de primeira (iStent) ou segunda geração (iStent *inject*). O Grupo 1 consistiu em olhos com pressão intraocular medicada <18 mmHg e o Grupo 2 consistiu em olhos com pressão intraocular medicada ≥18 mmHg. Os principais desfechos foram as taxas de sucesso relativo (com ou sem medicamentos) e completo (sem medicamentos) em diferentes pressões intraoculares-alvo, redução média (%) no uso de medicamentos e proporção de olhos sem medicamentos.

**Resultados:**

A média de idade foi de 70,4 anos no Grupo 1 (n=105) e 68,1 anos no Grupo 2 (n=65). As taxas de sucesso relativo para pressão intraocular <18 mmHg, pressão intraocular <15 mmHg e pressão intraocular <12 mmHg foram semelhantes entre os grupos (Grupo 1: 96,2%, 88,6% e 32,4%, respectivamente; Grupo 2: 93,8%, 78,5% e 21,5%, respectivamente; todos p>0,05). As taxas de sucesso completo foram significativa mente maiores no Grupo 1 do que no Grupo 2: pressão intraocular <18 mmHg (76,2% vs. 47,7%); pressão intraocular <15 mmHg (73,3% vs. 40%); pressão intraocular <12 mmHg (14,3% vs. 4,6%). A redução média no uso de medicamentos foi maior no Grupo 1 do que no Grupo 2. Ao final do acompanhamento, 79,0% dos olhos do Grupo 1 e 47,7% dos olhos do Grupo 2 estavam livres medicamentos.

**Conclusões:**

Ambos os grupos mostraram altas taxas de sucesso relativo, mas olhos com pressão intraocular medicada pré-operatória <18 mmHg apresentaram taxas de sucesso completo mais elevadas, bem como maiores chances de se tornarem livres de medicamentos para glaucoma.

## INTRODUCTION

There are two major groups of microinvasive glaucoma surgery (MIGS) techniques, i.e., those aiming to achieve rehabilitation of the natural aqueous outflow pathway (trabecular ablation, trabecular bypass, or Schlemm’s canal dilation) and those creating an artificial drainage pathway (subconjunctival or suprachoroidal drainage techniques), which should be reserved for eyes where the natural trabecular outflow system is no longer viable^(1,2,3,4)^.

The earlier the glaucoma stage is, the greater is the probability of a viable and functional posttrabecular outflow system^(4,5,6)^. Eyes that show a good response to intraocular pressure (IOP)-lowering medications or selective laser trabeculoplasty tend to have better viability of the natural aqueous outflow system^(4,5,6)^.

Trabecular microbypass devices, such as iStent and iStent *inject*, are used to create a direct passage through the trabecular meshwork, leading to communication between the anterior chamber and the posttrabecular structures^(3,7,8,9,10)^.

In open-angle glaucoma, surgeries are often indicated when medication treatment or laser trabeculoplasty has failed. This is probably not the best scenario for trabecular microbypass surgeries, as a failure of noninvasive therapies can be an indirect predictor of poor viability of the posttrabecular outflow system^(11)^.

We hypothesized that the best outcomes with trabecular bypass surgeries would be achieved when IOP is still under control with topical medications, as this is a sign of a viable and still functioning posttrabecular outflow system.

To the best of our knowledge, no clinical evidence is available regarding differences in trabecular microbypass surgery outcomes based on IOP control under glaucoma medications before surgery. Konopinska et al. prospectively assessed the success of combined cataract and first-generation trabecular microbypass procedure in eyes with a baseline IOP (after washout) above or below 26 mmHg without pharmacological therapy. They found that eyes with IOP<26 mmHg without pharmacological therapy achieved higher rates of success^(12)^.

This study investigated the outcomes of first- and second-generation trabecular microbypass devices as replacement therapy to medications in pharmacologically controlled (IOP <18 mmHg) compared to pharmacologically uncontrolled (IOP ≥18 mmHg) open-angle glaucoma patients.

## METHODS

This retrospective study examined eyes treated with either iStent or iStent *inject* in a single center.

The inclusion criteria were first- or second-generation trabecular bypass implant surgery, age >18 years, open-angle glaucoma (primary open-angle glaucoma, pigmentary glaucoma, or pseudoexfoliative glaucoma), glaucoma-only or combined with cataract surgery, at least 6 months of follow-up, and no ocular comorbidity. We included only one eye per patient; hence, if both eyes of the same patient were eligible, we randomly selected one of them.

The exclusion criteria were missing data in the records, all other types of glaucoma, including normal-tension glaucoma, and ocular comorbidity. We also excluded the first 10 cases of iStent implantation and the first five cases of iStent *inject* implantation because they were considered part of the learning curve. All surgeries were performed by the same surgeon in a single site.

In addition to the demographic and clinical data (age, sex, race, type of glaucoma, and glaucoma stage), we evaluated the mean follow-up time, changes in IOP, number of glaucoma medications, and visual acuity from the preoperative period to the end of follow-up.

The glaucoma stage was determined according to the Hodapp-Parrish-Anderson criteria based on the mean deviation (MD) of Humphrey computerized perimetry. Eyes were categorized as having mild (MD better than -6 dB), moderate (MD between -6.00 dB and -12.00 dB), and advanced (MD worse than -12.00 dB) glaucoma^(13)^.

The study population was divided into two groups: Group 1 (pharmacologically controlled IOP; baseline medicated IOP<18 mmHg) and Group 2 (pharmacologically uncontrolled IOP; baseline medicated IOP ≥18 mmHg). The main outcomes measured were reduction of the mean IOP (%), reduction of the mean number of medications (%), the proportion of medication-free eyes at the end of follow-up, qualified success rates (with adjunctive glaucoma medications as needed) at different IOP levels (IOP <18 mmHg, <15 mmHg, and <12 mmHg), unqualified or complete success rates (without adjunctive glaucoma medications) at different IOP levels (IOP <18 mmHg, <15 mmHg, and <12 mmHg), and probability of success determined by survival analyses based on two criteria (IOP <18 mmHg and IOP <15 mmHg). We also evaluated the numbers of intra- and postoperative complications.

All numerical variables were tested for normality of their distribution through the Kolmogorov-Smirnov test. Student’s *t* test was used for comparisons of variables with a normal distribution. Non-parametric tests (Kruskal-Wallis or Mann-Whitney test) were used for variables with a non-normal distribution. The chi-square test was used for analyses of categorical variables.

All statistical analyses were performed using IBM SPSS Statistics 25 (IBM Corp., Armonk, NY, USA). In all analyses, p<0.05 indicated statistical significance.

This study was performed in accordance with the Declaration of Helsinki and was approved by the Ethics Committee of the Santa Casa de Misericordia de Juiz de Fora (CAAE: 21327319.5.0000.5139).

## RESULTS

The study population consisted of 170 eyes with a mean ± standard deviation (SD) follow-up time of 20.1 ± 8.6 months (range 6-38).

Group 1 and Group 2 consisted of 105 eyes (61.8%) and 65 eyes (38.2%), respectively. The mean (± SD) follow-up periods were 21.2 (± 8.2) months and 18.4 (± 9.1) months, respectively (p=0.048). Table 1 presents the baseline characteristics of the groups. The baseline number of medications, glaucoma stage, and type of surgery were statistically different between groups and as these variables could have influenced our findings, we performed additional analyses for their control.

**Table 1.** Baseline characteristics of each group

Characteristics	Group 1 n=105	Group 2 n=65	p-value
Age (mean ± SD), years	70.4 ± 8.9	68.1 ± 10.5	0.132a
Baseline IOP (mean ± SD), mmHg	14.3 ± 2.0	19.9 ± 1.9	<0.001a
Baseline number of medications (mean ± SD)	1.9 ± 0.9	2.3 ± 1.0	0.004a
Race			
Caucasian	84.8%	83.1%	0.746b
African descent or mixed	15.2%	16.9%	
Sex			
Male	39.0%	44.6%	0.289b
Female	61.0%	55.4%	
Glaucoma stage			
Mild	87.6%	66.2%	<0.001b
Moderate	5.7%	27.7%	
Advanced	6.7%	6.2%	
Laterality			
OD	51.4%	53.8%	0.441b
OS	48.6%	46.2%	
Type of glaucoma	85.7%	83.1%	0.399b
POAG	14.3%	16.9%	
Other OAG			
Baseline visual acuity	61.0%	67.7%	0.526b
20/30 or better	34.3%	26.2%	
20/40 to 20/100	4.8%	6.2%	
20/200 or worse			
Type of surgery	87.6%	67.7%	0.002b
Combined with cataract	12.4%	32.3%	
Standalone			
Type of bypass	34.3%	23.1%	0.083b
iStent	65.7%	76.9%	
iStent *inject*			

IOP= intraocular pressure; OAG= open-angle glaucoma; OD= right eye; OS= left eye; POAG= primary open-angle glaucoma; SD= standard deviation.

^a^Student’s *t* test; ^b^Chi-square test

### IOP results

**Figure 1.**
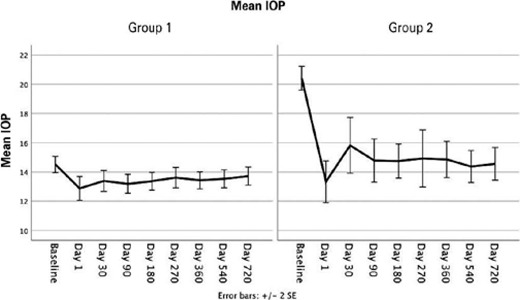
Changes in IOP from baseline to the end of follow-up for Group 1 (baseline IOP <18 mmHg under pharmacological treatment) and Group 2 (baseline IOP ≥18 mmHg under pharmacological treatment).

At the end of the follow-up, the mean IOP reduction (percentage) was 6.3% in Group 1 and 29.1% in Group 2 (between-group comparison, p<0.001). Figure 1 shows the changes in IOP for each group. Table 2 shows the changes in the mean IOP and mean number of glaucoma medications from baseline to 24 months and the sample size for each group during follow-up. Success rates according to IOP are given in table 3.

IOP= intraocular pressure.

**Table 2.** Evolution of mean IOP and mean number of glaucoma medications from baseline to month 24

Time point	Group 1		Group 2		Comparisons between groups for IOP^a^	Comparisons between groups for Meds^a^
	IOP (mmHg)	Meds	IOP (mmHg)	Meds		
Baseline	14.3	1.9	19.9	2.3	< 0.001	0.004
	n=105		n=65			
Day 1	13.3*	0.1*	12.9*	0.2*	0.523	0.787
	n=105		n=65			
Day 15	14.0	0.2*	16.4*	0.5*	0.002	0.005
	n=105		n=65			
Day 30	13.3*	0.2*	15.2*	0.8*	0.001	<0.001
	n=105		n=65			
Day 90	13.2*	0.2*	13.8*	0.8*	0.238	<0.001
	n=105	n=65	
Day 180	13.3*	0.3*	14.0*	0.8*	0.090	<0.001
	n=105		n=65			
Day 270	13.6*	0.3*	14.1*	0.8*	0.354	<0.001
	n=98	n=56	
Day 360	13.6*	0.3*	14.5*	0.9*	0.056	<0.001
	n=89		n=52			
Day 540	13.7*	0.3*	14.1*	0.9*	0.266	0.002
	n=78	n=33	
Day 720	13.7*	0.3*	14.6*	0.9*	0.154	0.007
	n=49		n=27			

*p<0.05 vs. baseline (paired-samples Student’s *t* test).

^a^Student’s *t* test for independent samples.

IOP, intraocular pressure; Meds: number of glaucoma medications per eye.

**Table 3.** Qualifed (with glaucoma medications) and unqualifed (without glaucoma medications) success rates for diferent IOP levels

Success criteria		Group 1 n=105	Group 2 n=65	p-value
IOP<18 mmHg	Qualified	96.2%	93.8%	0.363
	Unqualified	76.2%	47.7%	<0.001
IOP<15 mmHg	Qualified	88.6%	78.5%	0.061
	Unqualified	73.3%	40.0%	<0.001
IOP<12 mmHg	Qualified	32.4%	21.5%	0.087
	Unqualified	14.3%	4.6%	0.037

IOP= Intraocular pressure.

**Figure 2.**
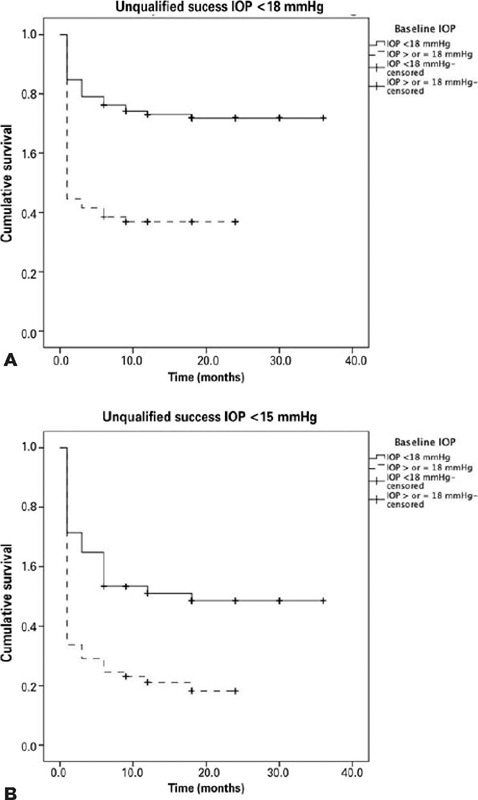
Kaplan-Meier survival curves for complete success for both groups: (A) IOP<18 mmHg and (B) IOP<15 mmHg.

We also performed survival analyses based on the Kaplan-Meier survival curves (Figure 2). The mean survival periods were significantly higher in Group 1 at both IOP <18 mmHg and IOP <15 mmHg with no medications. The probabilities of success at IOP <18 mmHg without medications were 72.4% and 36.9% for Groups 1 and 2, respectively. In addition, considering IOP <15 mmHg with no medications, the probabilities of success were 49.5% for Group 1 and 20.0% for Group 2.

### Reduction of medications

At the end of the follow-up, the mean reduction in the number of medications was 84.2% in Group 1 and 60.9% in Grroup 2 (between-group comparison, p=0.002).

Preoperatively, 58.1% and 78.5% of eyes in Groups 1 and 2 were receiving two or more glaucoma medications. At the end of follow-up (mean: 20.1 months), 79.0% of eyes in Group 1 and 47.7% in Group 2 became medication-free.

Table 2 summarizes the changes in the mean number of medications per eye in each group. The difference in the postoperative mean number of medications vs. baseline was statistically significant at all time points (p<0.001) for both groups.

**Figure 3.**
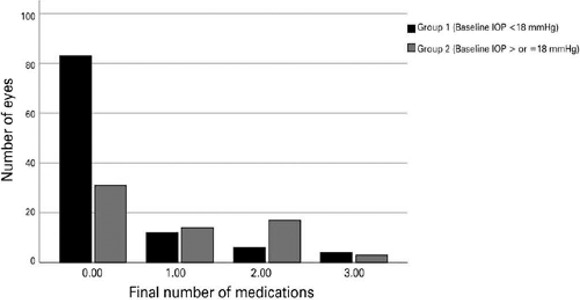
Proportion of eyes according to the number of glaucoma medications at the fnal time point for both groups.

Figure 3 shows the final number of glaucoma medications per eye at the end of follow-up (mean: 20.1 months).

### Visual acuity and safety results

Overall, 61% and 67.7% of eyes in Groups 1 and 2 (p=0.526), respectively, showed visual acuity of 20/30 or better at baseline whereas 90.5% and 96.9% of eyes in Groups 1 and 2, respectively, showed the same visual acuity level at the end of follow-up (p=0.263).

Both groups showed a high safety profile. Most patients (96.2% in Group 1 and 98.5% in Group 2) did not present any procedure-related complications intraoperatively (p=0.626). There were three cases (2.9%) of intraoperative excessive blood reflux in Group 1 and one case (1.5%) in Group 2. There was one case of iStent misplacement in group 1 (1.0%) and none in Group 2 (p=0.626).

Complications in the postoperative period were also rare. Most eyes in both groups did not have complications (98.1% of eyes in Group 1 and 96.9% in Group 2, p=0.438).

In Group 1, one eye showed peripheral anterior synechia occluding the internal ostia of the iStent, which was corrected by Nd:YAG laser iridotomy. In this group, one eye presented with uncontrolled IOP, requiring surgery during the follow-up. The patient could not tolerate any ocular hypotensive agents due to ocular allergy, and the iStent was not sufficient to control IOP.

In Group 2, two eyes required surgery due to uncontrolled IOP. These eyes developed steroid-induced IOP spikes. Patients had to be kept on systemic steroids for pulmonary and rheumatological diseases. These eyes were excluded from the analyses of IOP, medications, and success rates.

### Controlling for possible confounding variables

We performed univariate analysis for each possible confounding variable (baseline number of medications, glaucoma stage, and type of surgery) and a multivariate Cox regression survival analysis. Although Groups 1 and 2 were well-balanced according to the type of bypass (iStent vs. iStent *inject*), we decided to include this variable in this analysis.

#### Baseline number of medications

The mean number of baseline glaucoma medications was significantly greater in Group 2 than Group 1 (2.3 vs. 1.9, respectively, p=0.004). We excluded eyes receiving one medication at baseline from the analysis and only considered eyes with two or more medications at baseline in each group. Then, the mean number of medications was similar between the groups, along with the proportions of eyes on two, three, or four medications at baseline. Follow-up periods were also similar. However, the final mean number of medications was significantly higher in Group 2 than in Group 1 (1.2 vs. 0.5 medications, respectively, p=0.001), as was the number of eyes with no medications at the end of follow-up (67.2% vs. 33.3%, respectively, p<0.001).

In addition, analyses of qualified and complete success rates confirmed the outcomes described previously. The groups had similar qualified success rates but Group 1 had a higher complete success rate.

#### Glaucoma stage

Group 2 had a higher proportion of eyes with moderate glaucoma, and Group 1 had more eyes with mild glaucoma. We controlled for the glaucoma stage by evaluating outcomes considering only patients with mild glaucoma. In all, 92 were included in Group 1 and 43 in Group 2, and 84.8% and 60.5% of eyes, respectively, were medication-free at the end of follow-up. Qualified success rates were similar between the groups, however, eyes in Group 1 had a higher complete success rate, confirming our previous results.

#### Type of surgery (combined vs. standalone)

The results did not change when we controlled for the type of surgery. When we included only combined surgeries, more eyes were medication-free at the end of follow-up in Group 1 than in Group 2 (85.9% vs. 56.8%, respectively, p<0.001). There were no differences between the two groups in qualified success rates whereas complete success rates were significantly higher in Group 1.

#### Type of trabecular microbypass implant (iStent vs. iStent inject)

Considering eyes treated with iStent *inject* only, Groups 1 (n=69) and 2 (n=50) were similar according to age, race, baseline number of medications, sex, laterality, glaucoma type, type of surgery, and follow-up period. Again, the qualified success rates did not differ between the groups whereas complete success rates were significantly higher in Group 1. A significantly greater number of eyes treated with iStent *inject* were not receiving medications at the end of follow-up in Group 1. The percentage of eyes with IOP reduction was greater in Group 2 while the percentage of eyes with a reduction in the number of medications was higher in Group 1.

#### Multivariate Cox regression survival analysis

We considered IOP<18 mmHg without pharmacological treatment as a dependent variable for the regression model. All possible confounding or independent variables were included. The following independent variables were tested in the model: age, race, sex, laterality, baseline visual acuity, baseline number of medications, glaucoma stage, intra- and postoperative complications, type of surgery, and type of trabecular bypass (iStent or iStent inject). A significance of 95% was necessary for the variable to remain in the model.

Four steps were necessary until the final model was generated. Multivariate analysis confirmed baseline IOP<18 mmHg as a strong predictor of unqualified success, along with combined surgery and use of iStent *inject*. We present the regression results as well as their interpretation in table 4.

**Table 4.** Multivariate Cox regression survival analysis

Variable	Significance	Exp(β)	Interpretation
Baseline medicated IOP <18 mmHg	0.026	1.636	Presence of this variable is associated with 64% higher chance of achieving unqualified success
Combined Surgery	0.001	2.287	Presence of this variable is associated with 1.3x higher chance of achieving unqualified success
iStent *inject*	0.034	1.635	Presence of this variable is associated with 64% higher chance of achieving unqualified success

Final model (Step 4); backward stepwise method (likelihood ratio)

## DISCUSSION

The groups had similar qualified success rates for different IOP levels. However, medically controlled eyes at baseline (medicated IOP<18 mmHg) showed consistently higher rates of medication-free outcomes. Hence, trabecular microbypass is useful and effective for both situations. However, there is a greater likelihood of achieving a medication-free outcome for eyes with a baseline medicated IOP<18 mmHg. Therefore, for such eyes, trabecular stents are a very useful and effective tool as replacement therapy for medications.

Survival analyses showed that eyes in Group 1 remained medication-free significantly longer with a higher probability of success than eyes in Group 2. The chance of achieving no need for glaucoma medications was much lower at baseline IOP ≥18 mmHg under pharmacological treatment (Group 2), demonstrating that post-trabecular status is not the same as for eyes in Group 1. Trabecular stenting is still valuable, as all eyes achieved good success rates, although with the need for adjunctive use of glaucoma medications. These results suggest that stents can rehabilitate posttrabecular outflow to a certain degree. This may be because trabecular stents can rehabilitate the trabecular pathway in the inferonasal quadrant, but in eyes with a more advanced trabecular disease, rehabilitating only one quadrant helps but may not be enough to control IOP without the need for glaucoma medications.

Our results suggest two distinctive goals for trabecular surgery in the two study groups. For patients with baseline IOP <18 mmHg under pharmacological treatment, the goal of surgery may be very effective IOP control and no need for medications. Conversely, in patients with baseline IOP ≥18 mmHg under pharmacological treatment, the main goal is to reduce IOP and patients should be advised about the greater probability of requiring some glaucoma medications after treatment.

The observed reductions in IOP are consistent with other studies regarding iStent and iStent *inject*^(14,15,16,17)^. We found that IOP decreased by a mean of 16.5% for the whole cohort, which included both pharmacologically controlled and uncontrolled eyes. Using a baseline IOP cutoff point of 18 mmHg to separate controlled from uncontrolled eyes under pharmacological therapy, we found that the percentage of eyes that achieved IOP reduction was higher in those with higher (IOP ≥18 mmHg under pharmacological treatment) than lower baseline IOP (29.1% vs. 6.3%, respectively), consistent with Ferguson et al.^(18)^.r The higher is the baseline IOP, the higher is the percentage reduction in IOP. Therefore, there is a floor effect on the capacity of trabecular bypass for IOP reduction, which is the posttrabecular resistance to aqueous outflow. Regardless of the baseline IOP, there is always a greater chance of achieving a low to mid-teen final IOP value. If the eyes already have IOP close to the final reachable IOP value, based on the posttrabecular resistance, there is almost no additional reduction in IOP, and the benefit is in the reduction of medications.

The reduction of medication burden differed according to the baseline IOP under pharmacological treatment. The percentage of eyes with medication reduction was lower for those with IOP ≥18 mmHg than IOP <18 mmHg under pharmacological treatment (60.9% vs. 84.2%, respectively). More eyes in Group 1 did not completely need glaucoma medications at the end of follow-up than in Group 2 (79.0% vs. 47.7%). This difference between pharmacologically controlled and uncontrolled eyes could be explained by the viability and function of posttrabecular structures (i.e., Schlemm’s canal and collectors’ channels). When posttrabecular structures are preserved and still functional, there is a greater chance that only bypassing the trabecular tissue will be sufficient for IOP control. Our results could represent additional indirect evidence that IOP control with pharmacological treatment can be used as a reasonable surrogate for posttrabecular status and function.

Although assessing a different trabecular device, but which also acts by bypassing the trabecular meshwork, Fea et al. found that in patients with preoperative IOP of 18 mmHg or higher, the reduction in IOP and in the number of medications was higher^(19)^. The relationship between baseline IOP and the amount of IOP reduction is consistent in the literature, and we have also found that a higher baseline IOP is associated with higher reductions in IOP. However, outcomes concerning the relationship between the level of baseline IOP and the amount of medications reduction are contradictory. This could be related to differences in the study population and significant differences between the devices (size, mechanism of action, etc.).

As demonstrated in many previous studies, trabecular stenting procedures have a very good safety profile^(14,15,16,20)^. Most eyes showed improvement in visual acuity and were complication-free in both groups. No serious adverse events were observed during the follow-up.

Although the study population consisted of 170 eyes, the groups were not significantly similar in some important variables, such as baseline number of medications, glaucoma stage, and type of surgery (standalone or combined). These are important confounding variables, as they can also be a reflection of posttrabecular viability^(11)^. A smaller number of baseline medications, early glaucoma stages, and combined surgery are all related to a potentially better posttrabecular status^(11)^. Another possible confounding variable was the type of trabecular bypass device. iStent and iStent *inject* have different designs and slightly different efficacy profiles^(9,17,21)^. After controlling for such variables in both univariate and multivariate regression analyses, the results were consistent and robust, confirming that IOP level under pharmacological therapy was a strong driver for our outcomes.

The strengths of our study include a large sample size (n=170), long-term follow-up (6-38 months), a real-world setting, and a single center design. We also included both standalone and combined procedures, different glaucoma stages (mild to advanced stages), and first- and second-generation trabecular bypass devices.

However, our study had some limitations including its retrospective design. In addition, the majority of our patients were Caucasian, which limits the external validity of our findings. We have not controlled our study groups for medication adherence. This could have had an influence in our results, as some patients might have been included in group 2 (uncontrolled IOP) as non-respondent to medications, but the real issue could have been poor adherence. This confounding variable could have exacerbated the medication-free results in group 2, and we speculate that if we control for this important variable, the difference between groups would be even greater than we have found. When controlling for the number of baseline medications, we excluded patients on 1 medication. Results for patients under at least 2 medications might not be completely applicable for the patients on 1 medication, so one should be cautious when interpreting this attempt to control for such variables.

Further investigations, preferably in prospective and randomized clinical trials, are needed to confirm our findings.

In summary, both eyes with IOP<18 mmHg and IOP ≥18 mmHg under pharmacological treatment had high qualified success rates. However, eyes that were pharmacologically controlled at baseline (IOP<18 mmHg under pharmacological treatment) showed higher long-term complete success rates and greater chances of achieving no need for glaucoma medications postoperatively.
